# Complementation of the embryo-lethal T-DNA insertion mutant of *AUXIN-BINDING-PROTEIN 1* (*ABP1*) with *abp1* point mutated versions reveals crosstalk of ABP1 and phytochromes

**DOI:** 10.1093/jxb/eru433

**Published:** 2014-11-11

**Authors:** Yunus Effendi, Noel Ferro, Corinna Labusch, Markus Geisler, Günther F. E. Scherer

**Affiliations:** ^1^Leibniz Universität Hannover, Institut für Gartenbauliche Produktionssysteme, Abt. Molekulare Ertragsphysiologie, Herrenhäuser Str. 2, D-30419 Hannover, Germany; ^2^University of Bonn, Mulliken Center for Theoretical Chemistry, Institute for Physical and Theoretical Chemistry, Beringstr. 4, D-53115 Bonn, Germany; ^3^University of Fribourg, Department of Biology - Plant Biology, Chemin de Museé 10, CH-1700 Fribourg, Switzerland; ^4^Al Azhar Indonesia University, Department of Biology, Sisingamangaraja, Jakarta 12110, Indonesia

**Keywords:** AUXIN-BINDING PROTEIN1 (ABP1), *abp1 mutants*, early auxin-regulated genes, early light-regulated genes, gravitropism, phototropism, phytochrome, hypocotyl elongation, shade avoidance, *Arabidopsis thaliana*.

## Abstract

The function of the extracytoplasmic AUXIN-BINDING-PROTEIN1 (ABP1) is largely enigmatic. We complemented a homozygous T-DNA insertion null mutant of *ABP1* in *Arabidopsis thaliana* Wassilewskia with three mutated and one wild-type (wt) *ABP1* cDNA, all tagged C-terminally with a strepII–FLAG tag upstream the KDEL signal. Based on *in silico* modelling, the *abp1* mutants were predicted to have altered geometries of the auxin binding pocket and calculated auxin binding energies lower than the wt. Phenotypes linked to auxin transport were compromised in these three complemented *abp1* mutants. Red light effects, such as elongation of hypocotyls in constant red (R) and far-red (FR) light, in white light supplemented by FR light simulating shade, and inhibition of gravitropism by R or FR, were all compromised in the complemented lines. Using auxin- or light-induced expression of marker genes, we showed that auxin-induced expression was delayed already after 10min, and light-induced expression within 60min, even though TIR1/AFB or phyB are thought to act as receptors relevant for gene expression regulation. The expression of marker genes in seedlings responding to both auxin and shade showed that for both stimuli regulation of marker gene expression was altered after 10–20min in the wild type and *phyB* mutant. The rapidity of expression responses provides a framework for the mechanics of functional interaction of ABP1 and phyB to trigger interwoven signalling pathways.

## Summary

AUXIN BINDING PROTEIN1 (ABP1) mutants have properties of auxin- and red light-signalling mutants. A novel concept for growth control by ABP1 and phytochromes is indicated by this functional link.

## Introduction

For many years the function(s) of AUXIN BINDING PROTEIN1 (ABP1) remained enigmatic. In earlier work, ABP1 functions were associated with the plasma membrane (Napier, 1995). Besides regulation of K^+^ channel activity and membrane potential, protein kinase activity, phospholipase A activity, calcium influx, and other very rapid responses were described, which all are too rapid to be initiated by transcription and protein biosynthesis. Instead, post-translational mechanisms are suggested to initiate these rapid responses. For these ABP1 is thought to function as an auxin receptor (Scherer, 2011).

A conditional *ABP1* mutant was created by expressing an antibody against ABP1 in the apoplast which suppressed ABP1 functions like leaf expansion, endomitosis, cell division, and cell expansion (David *et al.*, 2007; Braun *et al.*, 2008; Paque *et al.*, 2014), results verified with an inducible mutant (Jones *et al.*, 1998; Chen *et al.*, 2001*a*). The only known T-DNA insertion mutant of this gene proved to be embryo-lethal (Chen *et al.*, 2001b). The point mutation *abp1-5*, obtained by TILLING, was useful to uncover the interaction of ABP1, PIN proteins, and ROP/RIC signalling in protein trafficking (Robert *et al.*, 2010; Xu *et al.*, 2010). More detailed investigations using the heterozygous *ABP1*/*abp1* T-DNA insertion line revealed that functions like auxin-induced gene expression, phototropism and gravitropism, and auxin transport are defective in this mutant (Effendi *et al.*, 2011; Effendi and Scherer, 2011). Recently ABP1 has been linked to red light physiology, using *ABP1*/*abp1* and *abp1-5* (Effendi *et al.*, 2013), and to control of TIR1 activity (Effendi *et al.*, 2011; Tromas *et al.*, 2013).

Both *ABP1*/*abp1* and *abp1-5* have weak phenotypes so that progress in ABP1 research based on these mutants is still limited. On the other hand, the embryo lethality of a homozygous T-DNA insertion plant (Chen *et al.*, 2001b) opened up the possibility to complement this plant not only with wild-type but also with point-mutated cDNAs. We describe here such a series of mutants based on complementation of the knock-out plant that show more severe auxin-related phenotypes than previous *abp1* mutants. These results reveal that not only auxin but also phytochrome signalling is compromised in these lines.

## Material and methods

### Quantum chemical modelling

A theoretical examination of the geometry, electronic structure, and electronic binding energies (∆E) of the auxin binding pocket of ABP1 were performed. The structural data was obtained from the crystal structure of ABP1 (Protein Data Bank with accession codes 1LRH). The pocket containing the 1-NAA molecule and the surrounding amino acids at 6 Angstroms (~400 atoms) was isolated (amino acids: I22, L24, W44, Q46, I48, T54, P55, H57, H59, E63, F65, H106, V108, V121, I130, L132, F149, W151). The geometric structure of the wild-type pocket was optimized taking into account previous analysis of auxin molecules (Ferro *et al.*, 2006) and protein cavities (Rolo-Naranjo, *et al.*, 2010). The optimization was carried out using Density Function Theory (DFT) using the b3-lyp function (Becke, 1988, Lee *et al.*, 1988, Stephens *et al.*, 1994) including the Van der Waals correction D3 (Grimme *et al.*, 2010) and atomic basis sets at triple zeta level (def-TZVP) (Eichkorn *et al.*, 1997). The input geometry constrained 17 atoms in order to conserve the pocket structure and the start charge of the pocket was 2+ owing to the influence of Zn^2+^. All calculations have been performed with the program package TURBOMOLE (http://www.turbomole.com).

Different computational chemistry experiments were conducted to analyse the influence of mutations of the amino acids at positions 25, 54, 106, and 151 and the substitution of IAA in the position of 1-NAA. The substitution (mutants) H106 to N106, L25 to Y25, T54 to I54 were modelled and their geometries re-optimized at DFT level with b-lyp and the D3-correction. The re-optimizations included both pocket–auxin pairs and pockets alone to investigate ∆E. The ∆E energies were calculated by a single point calculation with b3-lyp and TZVP basis set following the equation: ∆E_bind_=∆E_pocket – aux_ – (∆E_pocket_ + ∆E_aux_) comparing each mutant with the wild type. The calculations solve the electronic problem accurately and, neglecting changes of pressure and volume in the cell, we hold that the electronic energy and the enthalpy are approximately equal (∆E=∆H). Our calculation will not allow for entropic processes according to the ∆G. For further analysis of the potential surface and electric field of the pocket we used the theory of deformed atoms in molecules (DAM; Rico *et al.*, 2004) as well as the comparison of electronic features using quantum similarity measures (Ferro *et al.*, 2006), applied now at pocket level using the auto values Z_AA_(Ω)=∫ρ_A_(r)Ω(r)ρ_A_(r)dr, where the operator at Ω were Coulomb and Overlap. This analysis offers details about the differences of the electronic features of each pocket.

### Plant material and growth conditions

*Arabidopsis thaliana* Wassilevskija (Ws) heterozygous wt plants containing a T-DNA insertion and kanamycin resistance were used for transformation. *ABP1* cDNA containing FLAG-tag and strep-tag II directly before the C-terminal KDEL under control of the 35S promoter was provided by T. Reinard (University of Hannover).This construct was then cloned into pENTR D-TOPO (Invitrogen) where site-directed mutation was performed using QuikChange™ Site-Directed Mutagenesis Kit (Stratagen). Entry vectors were cloned into destination vector pB2GW7 (basta resistance: Karimi *et al.*, 2002). The complete *ABP1* cDNA sequences in the vectors were sequenced after transformation into *Agrobacterium* and the designed mutations verified (MWG-Biotech AG Eurofins Genomics, Ebersberg, info-eu@eurofins.com). Confirmed vectors were used to transform *Arabidopsis thaliana* heterozygous *ABP1*/*abp1* plants (Chen *et al.*, 2001b). Progenies of the transformed plants were selected on agar plates containing kanamycin (50 µg/ml) and BASTA (30 µg/ml). Surviving seedlings were PCR genotyped to identify homozygous null ABP1 wt plants (primer list: see Supplementary Table S2). Double homozygous lines were selected from these.

Seedling experiments were performed on sterile 1% (w/v) agar (growth experiments), 0.5% (w/v) gelrite to stabilize tropism experiments (Santner and Watson, 2006), or liquid (seedlings for RNA extraction) half-strength Murashige and Skoog (MS) medium containing 1% (w/v) sucrose at 22 °C for 10 d or as otherwise indicated (Figs 2 and 3). Experiments were repeated two to three times independently (*n*=75–90).

Auxin sensitivity was repeated twice by transferring light-grown seedlings at day 4 to media containing increasing IAA concentrations (0–10 µM) or mock for further growth for 6 days (Figs 2D–F; 5L, M). Hypocotyl lengths in light-grown or auxin-treated seedlings were calculated by subtracting the lengths obtained without auxin (Fig. 5L, M). Dark-grown seedlings were pre-grown in liquid half-strength MS for two days without auxin (Fig. 5L). Auxin was added and the increments of hypocotyl lengths after 12h were determined. Basipetal auxin transport was measured according to Lewis and Muday (2009). Radioactive auxin was applied to the root tip and segments cut after 8h (5–10mm, 10–15mm, and 15–20mm from tip) and counted after 18h. The 5–10mm segment in the wt was set as 100% and others calculated accordingly (Fig. 2J).

Plants were cultured in soil on a growth chamber at 22 °C constant 8h/16h (light/dark; SD) on peat-based compost soil (Einheitserde, http://www.einheitserde.de/) containing 30% silica sand. Leaves were measured from the three largest leaves from each of 60 adult plants per genotype. Rosette leaf number at 59 d and flowering time was obtained in two independent replications (30 plants each). Flowering time (first flower with white petals) for each genotype was recorded. Apical dominance at 90–92 days was measured as the number of branches at the bottom of fully grown plants with 100 plants each grown in SD (Fig. 4H).

For seedling light experiments, seeds were stratified for 4 d, plates were placed in horizontal position at 22 °C under white light (W) for 2h before transfer for 1 d into darkness. Then they were kept for 3 d either in constant R, FR, or B (0.1 µmol m^–1^ s^–2^ or 1 µmol m^–1^ s^–2^) or dark (Fig. 5A–K). For shade avoidance experiments, seeds on plates prepared like as were exposed to 24.5 µmol m^–1^ s^–2^ constant white LED light for 3 d. Then to W either low R/FR ratio (0.098) or high R/FR ratio (2.1) was added for 3 d (spectra: see Effendi *et al.*, 2013). For RNA extraction, seedlings received the low red (LR) or high red (HR) treatment for 1h after 3 d in W (Fig. 7; Fig. 8). In experiments with NPA (naphthylpthalamic acid) this was added to the plates from the start of the experiment (Fig. 6). Data were obtained from three independent replications and each replication was consisted of more than 40 seedlings. Light experiments were done without sucrose in the medium in an LED chamber (CLF, Plant Climatics) (Effendi *et al.*, 2013) (Fig. 5). All quantifications were done by scanning the plates with CanonScan 8800F (resolution of 600 dots per inch; Canon, http://www.canon-europe.com) and evaluating lengths or angles with AXIOVISIOLE version 4.6 software (Zeiss, http://www.zeiss.com/) and analysed using the t-test in Excel.

### Nucleic acid analysis

Seedlings were grown on half-strength MS liquid media in W for 14 d for auxin treatment (for light treatments see above). Seedlings were then equilibrated for 2h in fresh half-strength MS liquid media and then 10 µM IAA or mock was added. qPCR and statistics were performed as described (Livak and Schmittgen, 2001; Pfaffl *et al.*, 2002; Effendi *et al.* 2013). Test gene and reference gene primers are listed in Supplementary Table S2.

## Results

### Modified auxin binding binding box in ABP1

**Fig. 1.**
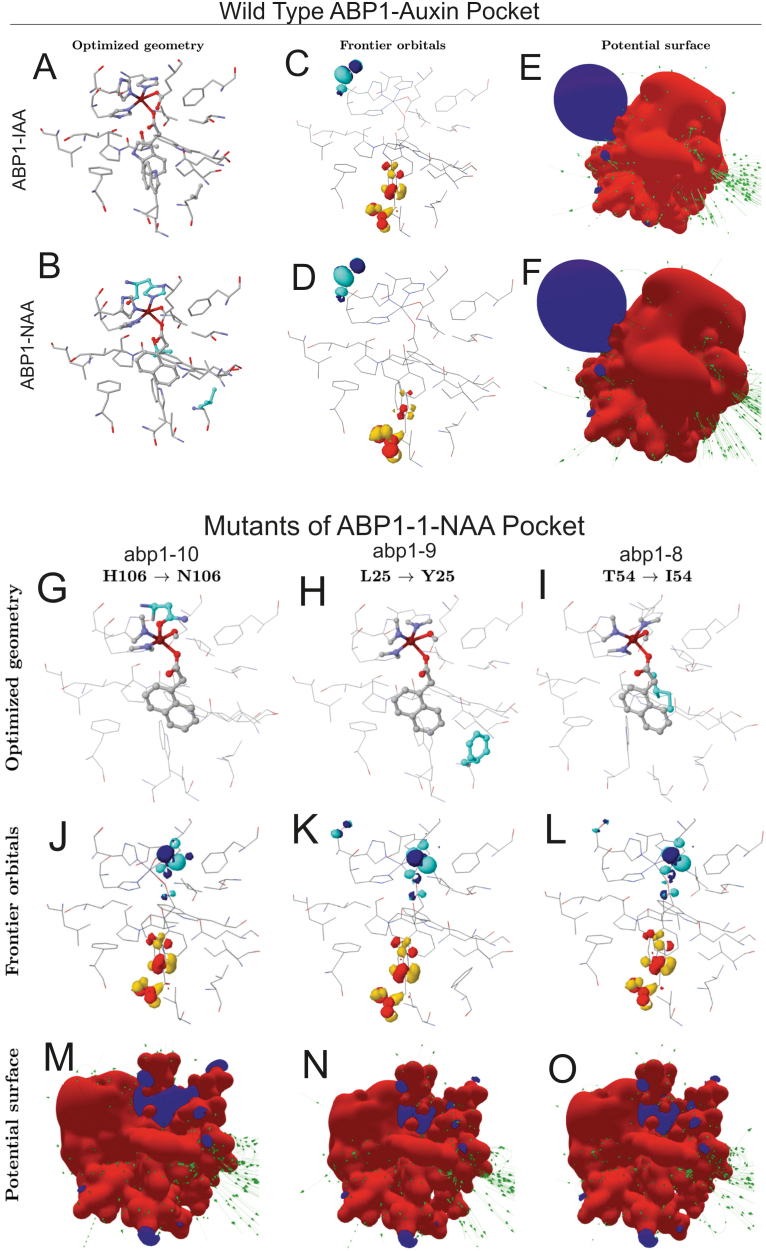
Modelling of changes in the geometry and electronic structure of the ABP1 pocket resulting from substitutions in the amino acid sequence. (A–F) The panels of the wild type ABP1-auxin binding pocket show optimized geometry (A, B), frontier orbitals (C, D), and potential surfaces (E, F). Mutated amino acids are highlighted in blue in panel B and individually in panels G–I. Note that in mutants not only geometries are changed (G–I) but also the geometries of the highest occupied molecular orbital [HOMO (red-yellow)] on W151 and the lowest unoccupied molecular orbital [LUMO (blue-cyan)] on H59 of the Zn^2+^ complex (K–N).

We designed and developed new *Arabidopsis abp1* mutants by transforming the kanamycin resistant *ABP1*/*abp1* mutant (Chen *et al.*, 2001b) with wild type (wt) *ABP1* cDNA or *ABP1* cDNA containing point mutations in the auxin-binding site of ABP1 (Woo *et al.*, 2002) (Fig. 1) using Basta selection. A strep II tag and a FLAG tag were inserted immediately upstream of the C-terminal ER retention motif KDEL. We were able to isolate four stable *abp1* mutants, *abp1-8* (T54>I54), *abp1-9* (L25>Y25), *abp1-10* (H106>N106), and *abp1-11* (no point mutation but tagged) in the background of the homozygous T-DNA insertion null mutant. The isolation showed that doubly resistant transformed T1 seedlings could be obtained and selected. Other lines did not propagate or produced very few doubly resistant plant progeny. From progeny of the mutant lines we theoretically expected one in four plants to have no wt ABP1 owing to homozygosity of the T-DNA insertion, but we needed to genotype 500–700 individuals until we found the desired mutant, still heterozygous for the basta marker. Selfing then gave lines homozygous for the basta marker. Owing to the difficulty in producing suitable lines we chose to consider those four orthologous lines as a set rather than isolating several lines of each mutant.

### Quantum chemical modelling

We concentrated the analysis on an accurate quantum chemical model using density functional theory (DFT) for describing geometry, chemical bonds, electronic properties, and electronic binding energies of the wild-type auxin-binding pocket of ABP1 and the three site-specific mutations. The optimized structures of all binding pockets (Fig. 1A, B, G–I) demonstrated that the coordination number of the Zn^2+^ atom was 5 with square-based pyramidal geometry (Alberts *et al.*, 1998), one of which coordinates the carboxyl group of the auxin ligand.

The electron donor-acceptor regions or frontier molecular orbitals (Fig. 1D, J–L) of the wt auxin pocket (Fig. 1C, D) play an important role to determine the activity of auxin and auxin-like molecules (Ferro *et al.*, 2006). The calculations of binding pocket geometry were complemented by visualizing the pocket surface (Fig. 1E, F). We determined the interaction to ligands by observing the frontier orbitals (HOMO, highest occupied molecular orbital; LUMO, lowest unoccupied molecular orbital; Fig. 1C, D). The electron donor W151 (wt) is plotted in yellow-red (HOMO) and the acceptor, concentrated around H59, is plotted in blue-cyan (LUMO) surrounding the Zn^2+^, and both are independent of the presence of 1-NAA or IAA. In the presence of either 1-NAA or IAA the localization of the frontier molecular orbitals is nearly identical and the surface pattern potentially exposed to ligand is strongly polarized (Fig. 1E, F). The region formed by the Zn^2+^ complex presents the negative potential (blue), and the remainder of the pocket is dominated by the positive potential (red) of other amino acids. Both results, the position of the frontier orbitals in the pocket and the polarized potential, are consistent. The green lines represent the electrostatic field lines or force produced by the atoms.

Calculations were done for the substitutions of the amino acids L25>Y25 (*abp1-9*), T54>I54 (*abp1-8*), and H106>N106 (*abp1-10*) in the wt structure of the auxin pocket of ABP1 (Fig. 1G–O). The polarization of the surface potential observed in the wt pocket was lost in the mutations (Fig. 1J–L). In addition, the HOMO–LUMO localization depicted (Fig. 1G–I) showed that every mutation changes the localization of the electron acceptor (LUMO) from the H59 to the E63 (Fig. 1J–L).

The changes in the Coulomb matrix have previously been connected with the biological activity of the auxin molecules and in binding specificity of auxin molecules (Ferro *et al.*, 2006). To correlate the physiological properties of the mutants with modelling, we focussed on further quantum chemical calculations of dE, Overlap, and Coulomb matrices. The electronic binding energies suggest that the mutants H106>N106 [*abp1-10*: –37.51 dE (Kcal/mol)], and T54>I54 [*abp1-8*: –35.90 dE (Kcal/mol)] offer less stability for binding the auxin molecule. Two mutants, *abp1-10* and *abp1-8*, also showed similar trends in the changes of the Overlap and Coulomb auto values (Supplementary Table S1) indicating similar binding properties. Though the calculated binding energies are similar in both the wt [–41.10 dE (Kcal/mol)] and *abp1-9* [–42.14 dE (Kcal/mol)], differences in Overlap and Coulomb matrices and geometry will influence binding because the smaller L25 is replaced by the bulky Y25, increasing electronic interaction with auxin similar to W151 (Woo *et al.*, 2002) but, at the same time, also restricting pocket space. Accordingly, the Coulomb auto value, representing the charge surface of the pocket for electrostatic interactions with the ligand, and the Overlap auto value, representing the electron density of the pocket surface, in *abp1-9* showed the strongest differences to the wt of these two parameters (Supplementary Table S1). Combined with the effects of dE this indicates a decrease of function in *abp1-9*. The strep tag is already outside of the main part of the ABP1 and thus most probably does not interfere with auxin binding (see Supplementary Fig. S1).

### Auxin-related functions are compromised and auxin sensitivity is lower in *abp1* mutants

**Fig. 2.**
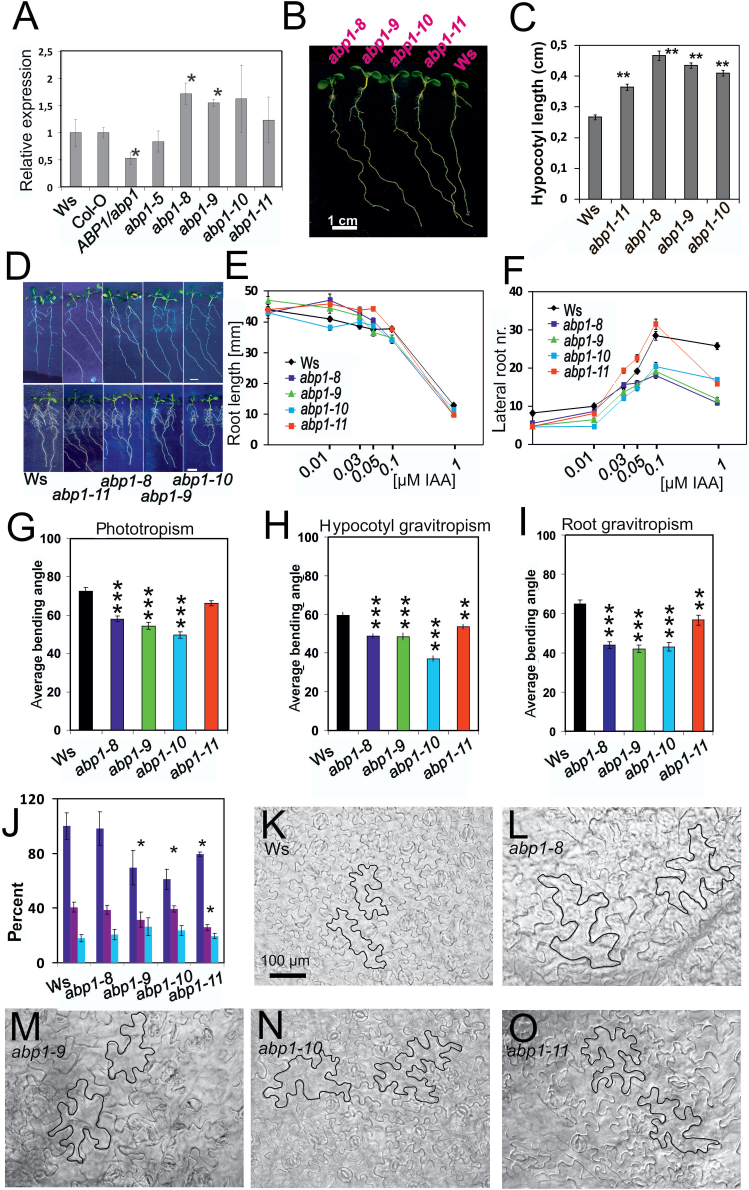
Developmental physiology of *abp1* mutant seedlings. (A) Level of *ABP1* transcript in *abp1* mutants in comparison to the respective wild types *ABP1* (*abp1-5* is in Col-O, all others in Ws). Value for wt *ABP1* is set as 1 (asterisk: different by *P*<0.05). (B) Representative light-grown seedlings (7 d; Ws, *abp1* mutants). Bar=5mm. (C) Hypocotyl length of light-grown seedlings (14 d) (*n*=23–30; SEM, *P*<0.01). (D) Root development without auxin (upper row) and in the presence of 0.1 µM IAA (lower row) (*n*=30–39; SEM). (E) Auxin sensitivity of primary root length and (F) and lateral root number (*n*=30–39; SEM). Error bars are either visible or smaller than symbols. Non-overlapping symbols or error bars in E and F were significantly different from each other (*P*<0.01 or lower). (G) Delayed phototropic responses of hypocotyls of dark-grown (3 d) seedlings. Phototropism was induced by lateral blue light (10 µmol m^–1^ s^–2^) for 8h (SEM, *n*=70–130). (H) Delayed gravitropic responses in hypocotyls of 3-day-old dark-grown seedlings after 24h tilting by 90° (*n*=63–140). (I) Delayed gravitropic responses of roots of dark-grown seedlings (3 d) after 24h tilting by 90° (*n*=52–90). (G–I; average bending angles±SEM * *P*<0.05; ** *P*<0.01; *** *P*<0.001). (J) Acropetal auxin transport in roots of 4-day-old light-grown seedlings. Dark blue bars: 5–10mm from tip; purple bars: 10–15mm from tip; light blue bars: 15–20mm from tip. (*n*=40; *: *P*<0.05). (K–O) Epidermal pattern of primary leaves. (K) Wassilewskia wt; (L) *abp1-8*; (M) *abp1-9*; (N) *abp1-10*; (O) *abp1-11.* In each photo two cells are outlined for comparison. Bars=100µm. Cell areas and lobe numbers of epidermis cells are presented in Supplementary Fig. S2.

The mutants transcribed the mutated *ABP1* genes at about 1–1.7-fold the level of the wt Ws (Fig. 2A). The *ABP1*/*abp1* mutant (Ws) was transcribed only at about 50% wt level and the *abp1-5* mutant (Col-0) at about 80% of the corresponding wt.

All four *abp1* mutants had longer hypocotyls (Fig. 2B, C) than wt, although in *abp1-11* this phenotype was modest. When *abp1* mutants were grown on auxin, *abp1-8*, *abp1-9*, and *abp1-11* roots were longer than wt roots. All other genotypes had lengths similar to the wt (Fig. 2D, E). In response to 0.03 µM or higher auxin, a clear decrease in lateral root number was found in *abp1-8*, *abp1-9*, and *abp1-10*, but not in *abp1-11* (Fig. 2D, F). These data indicated lower auxin sensitivities for the three *abp1* mutants.

Phototropic and gravitropic bending of hypocotyls and gravitropic bending of roots of *abp1* mutants was slower than for wt and *abp1-11* (Fig. 2G– I). Gravitropic bending of hypocotyls and roots of dark-grown *abp1* mutants was delayed (Fig. 2H, I) and bending angles of hypocotyls and roots were clearly smaller (Fig. 2H, I). Acropetal auxin transport from shoot base to root tip was delayed in all mutants except *abp1-8* (Fig. 2J).

Leaf cell growth and epidermal cell lobe numbers are ABP1-dependent (Xu *et al.*, 2010). Epidermal cells were larger in *abp1-8*, *abp1-9*, and *abp1-10* (Fig. 2K–N), but only weakly so in *abp1-11* (Fig. 2O; Supplementary Fig. S2D). Suppression of epidermal cell lobes per cell area was most pronounced in *abp1-8* and in *abp1-9* (Fig. 2L, M; Supplementary Fig. 2S). From these data we conclude that the three *abp1* site-directed mutants generally were less sensitive in their responses to auxin, or to responses involving auxin transport than wt. Such auxin-related properties were less prominent in *abp1-11*, which resembled more the wt, with the small phenotype possibly associated with the presence of the tag.

### Early expression of auxin-induced genes in *abp1* mutants is insensitive to auxin

**Fig. 3.**
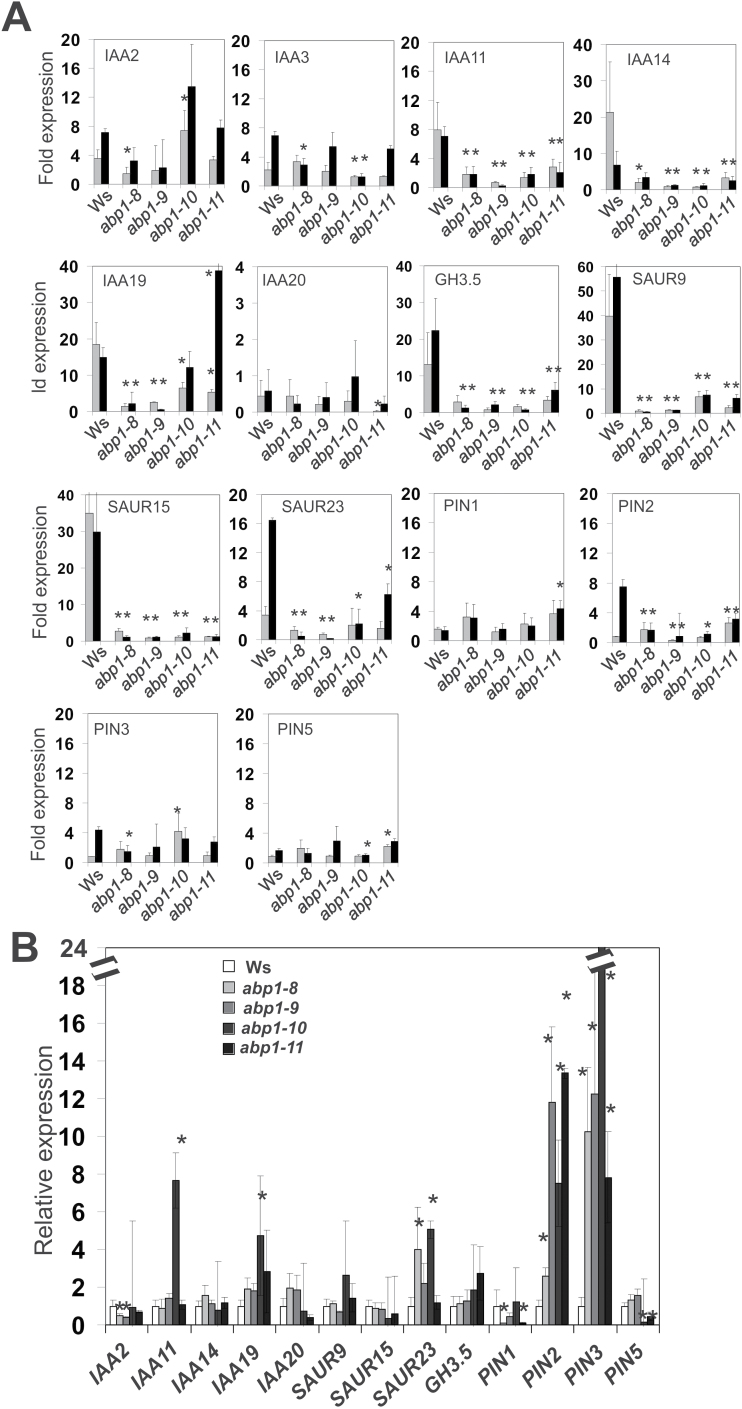
Expression of early auxin genes and several PIN genes in 14-day-old light-grown seedlings. Seedlings were either treated with 10 µM IAA in (A) (grey bars: 10min, black bars: 30min) or mock in (B). qRT-PCRs were from three biological replicates with three technical replicates for each gene. Statistical analysis was performed as described by Livak and Schmittgen (2001) and verified using the method of Pfaffl *et al.* (2002). At t=0min fold expression was set as 1 for the wt in (B). Asterisks indicate significant difference to the wt (* *P*<0.05; ** *P*<0.01).

We chose rapidly responding genes *IAA2*, *IAA3*, *IAA11*, *IAA14*, *IAA19*, *IAA20*, *GH3-5*, *SAUR9*, *SAUR15*, and *SAUR23* and *PIN* genes *PIN1*, *PIN2*, *PIN3*, and *PIN5* to test for the role of ABP1 on the control of gene expression (Effendi and Scherer, 2011; Effendi *et al*., 2011 2013 2014; Labusch *et al.* 2013). In the wt most marker genes were up-regulated after 10min of auxin application, whereas *IAA3*, *IAA20*, and the *PIN* genes were unchanged. After 30min wt expression of *PIN2* and *PIN3*, but not *PIN5* and *IAA20* were also up-regulated (Fig. 3A).

Auxin-induced expression of the marker genes was delayed in all four *abp1* mutants compared with the wt, with the exception of *IAA2*, *IAA3*, *PIN1*, *PIN3*, and *PIN5*. The greatest differences were found for the three *SAUR* genes, *GH3-5*, *IAA14*, and *IAA19*. Taken together, the delayed expression of auxin-induced marker genes clearly indicated insensitivity to auxin in *abp1-8*, *abp1-9*, and *abp1-10* compared with wt. Delayed expression was generally small in *abp1-11*.

### Morphology and flowering of adult plants

**Fig. 4.**
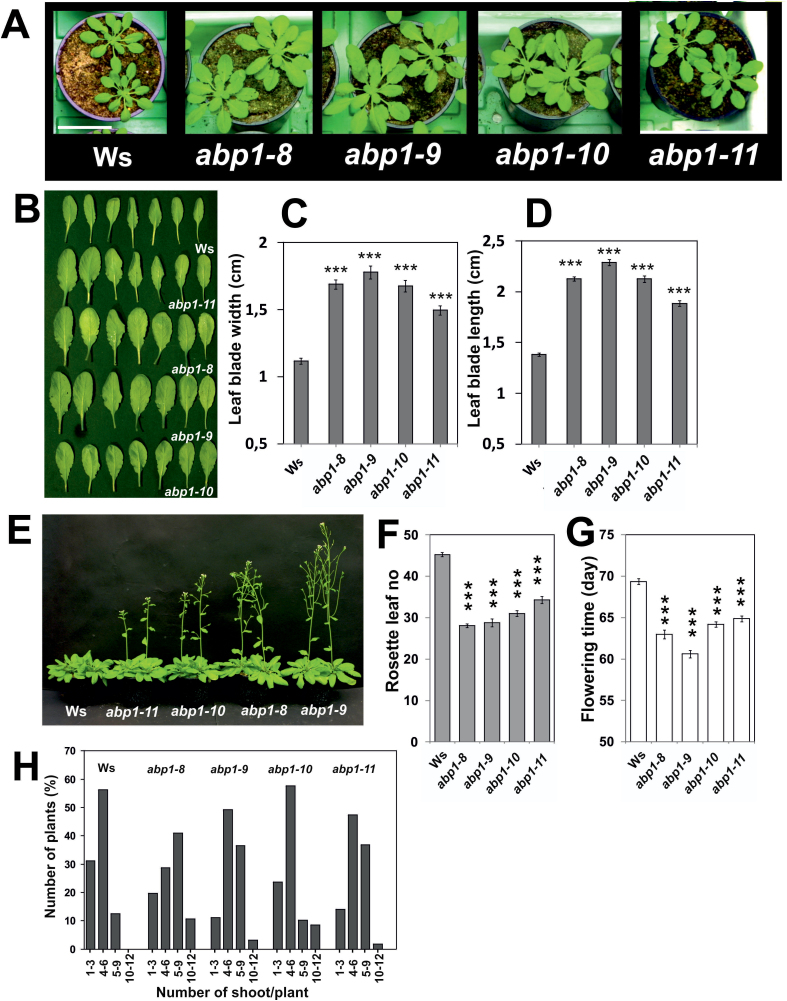
Phenotypes of *abp1* mutants grown in short days (8h/16h light/dark). (A) Representative images of 37-day-old rosettes of Ws and *abp1* mutants (*abp1-8*, *abp1-9*, *abp1-10*, *abp1-11*). Bar=5cm. (B) Representative images of leaves of plants shown in (A). (C) Leaf blade width and (D) blade length measured from 59-d-old plants (*n*=132–190; SEM; *P*<0.01). (E) Flowering plants at day 59 (*n*=30). (F) Rosette leaf number at flowering date (*n*=30). (G) Flowering time. Values (F, G) are means with SEM (*P*<0.001). Shown by asterisks is significance between the wt and mutants. (H) Apical dominance. Branches at the bottom were counted from 100 plants each at 90–92 d. [C, D, F, G: when error bars do not overlap values are significantly different from each other (*P*<0.05 or lower)].

The *ABP1*/*abp1* plants were in the Ws background (Chen *et al.*, 2001b) where a deletion in *phyD* renders this gene non-functional (Aukermann *et al*., 1997). The lack of a *phyD* gene influences early flowering (Effendi *et al.*, 2014). We observed that *abp1* mutants had longer and wider leaf blades (Fig. 4A–D), which is reminiscent of *phyA* mutants when grown under identical conditions (Supplementary Fig. S3). Flowering was earliest in *abp1-9* followed by *abp1-8* and *abp1-10* and finally the wt (Fig. 4E–G). In comparison to the wt *abp1-11* flowered early, but later than the other *abp1* mutants. In addition, we found decreased apical dominance in short days in *abp1-8 and abp1-9* lines, relatively weak decreases in *abp1-10* and *abp1-11*.

### *abp1* mutants have altered responses to continuous light and shade

**Fig. 5.**
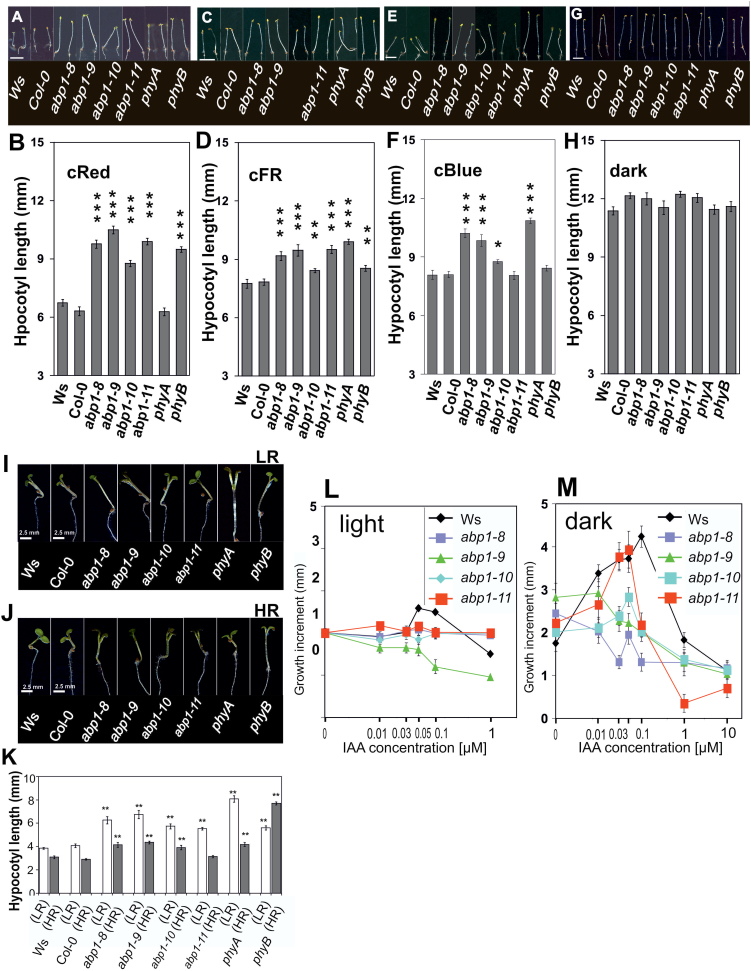
Elongation in monochromatic continuous light (R, FR, B) of 4 d-old seedlings in Ws and *abp1* mutants, *phyA* and *phyB*. (A, C, E, G) Representative images of seedlings grown in FR, R, B (0.1 µmol m^–1^ s^–2^ each) or dark, respectively. Bar=5mm. (B, D, F, H) Hypocotyl lengths (*n*>80; SEM). (I–K) Responses of hypocotyls of 3-d-old seedlings grown in W and then transferred for three more days to W with added low ratio R:FR (FR-enriched light) and high ratio R:FR (R-enriched light) in Ws wt, *abp1* mutants, and *phyA* and *phyB*. (I, J) Representative images of seedlings. (K) Hypocotyl lengths. (*n*>120; SEM). (B, D, F, H, K) Shown by asterisks is significance between the wt and mutants (*P<0.05; ** *P*<0.01; *** *P*<0.001). When error bars do not overlap values of different bars in one graph are significantly different from each other (*P*<0.05)). (L, M) Hypocotyl elongation in light (L) and dark (M) in the presence of increasing auxin concentrations. (L) Hypocotyl length increment induced by auxin of light-grown seedlings (see also Fig. 2A). (*n*=20–30, SEM). (M) Seedlings were grown in dark for 2 d and the length increment during subsequent 12h was recorded (*n*=20–30, SEM). Error bars are either visible or smaller than symbols. Data in L and M are significantly different between control and auxin-treated seedlings for each line when symbols or error bars do not overlap.

We investigated the growth of *abp1* mutants in continuous monochromatic FR, R, or blue (B) light and in darkness to test for the involvement of a photoreceptor (Fig. 5). In continuous R, all *abp1* seedlings had significantly longer hypocotyls than wt seedlings (Fig. 5A, B), similar to *phyB* but not to *phyA* seedlings. In continuous FR, all *abp1* mutants also displayed longer hypocotyls in comparison to wt seedlings, but were shorter than *phyA* seedlings (Fig. 5C, D). As hypocotyl elongation is inhibited by continuous FR in a fluence- and *PHYA*-dependent manner (Whitelam *et al.*, 1993) the data indicate that *abp1* mutants interfere with phyA-mediated responses. However, not all *phyA* deficiency responses in de-etiolated seedlings were observed in *abp1* mutants because they had opened and expanded cotyledons and displayed no apical hook, both responses not found in *phyA* seedlings (Fig. 5C). Hypocotyl elongation of *abp1* mutants displayed small differences under continuous B like *phyA* seedlings (Fig. 5E, F). Because of the small magnitude of B insensitivity of *abp1* seedlings, similar to *phyA* seedlings, assigning B insensitivity to either compromised phyA function or to insensitivity of a B receptor was not possible (Fankhauser and Casal, 2004). A dark phenotype was not obvious (Fig. 5G, H). Qualitatively similar but quantitatively smaller results were obtained when plants were grown in 1 µm^–1^ s^–2^ monochromatic light (see Supplementary Fig. S4).

In white light (W) a decrease in the R/FR ratio is the main cue for plants to perceive the presence of neighbours as physiological shade. W supplemented by a low ratio R:FR (LR) leads to strong elongation. W with added high ratio R:FR (HR) represses elongation. Responses to shade depend mainly on a low phyB signalling input (Fankhauser and Casal, 2004). Hypocotyl lengths in LR and HR were analysed (Fig. 5I–K). Seedlings of *abp1* mutants displayed significantly longer hypocotyls than wt under LR, whereas *abp1-11* seedlings were only slightly longer (Fig. 5I, K). Surprisingly, *abp1* mutants in HR also had hypocotyls longer than the wt, except *abp1-11* (Fig. 5J, K). This indicated that the strong *abp1* mutant alleles might be defective in phyB-mediated responses to physiological shade.

We tested the effect of auxin on light- and dark-grown seedlings. IAA applied in the light increased hypocotyl elongation slightly in the wt. In *abp1-9* auxin inhibited slightly and in *abp1-8*, *abp1-10*, and *abp1-11* it had no effect (Fig. 5L). However, exogenous IAA in 2-day-old dark-grown seedlings did stimulate elongation in wt and *abp1-11*, with an optimum at 0.05 µM IAA. This was not the case in *abp1-8*, *abp1-9*, or *abp1-10* (Fig. 5M) suggesting that ABP1 was a receptor for growth in dark-grown tissue. Reduced growth repression in R (Fig. 5A, B) in the *abp1* mutants was consistent with the observation that a fully functional ABP1 supports repression of growth in the light.

### NPA as an indicator for interaction of ABP1 and phytochromes in shade-induced elongation and inhibition of hypocotyl gravitropism

**Fig. 6.**
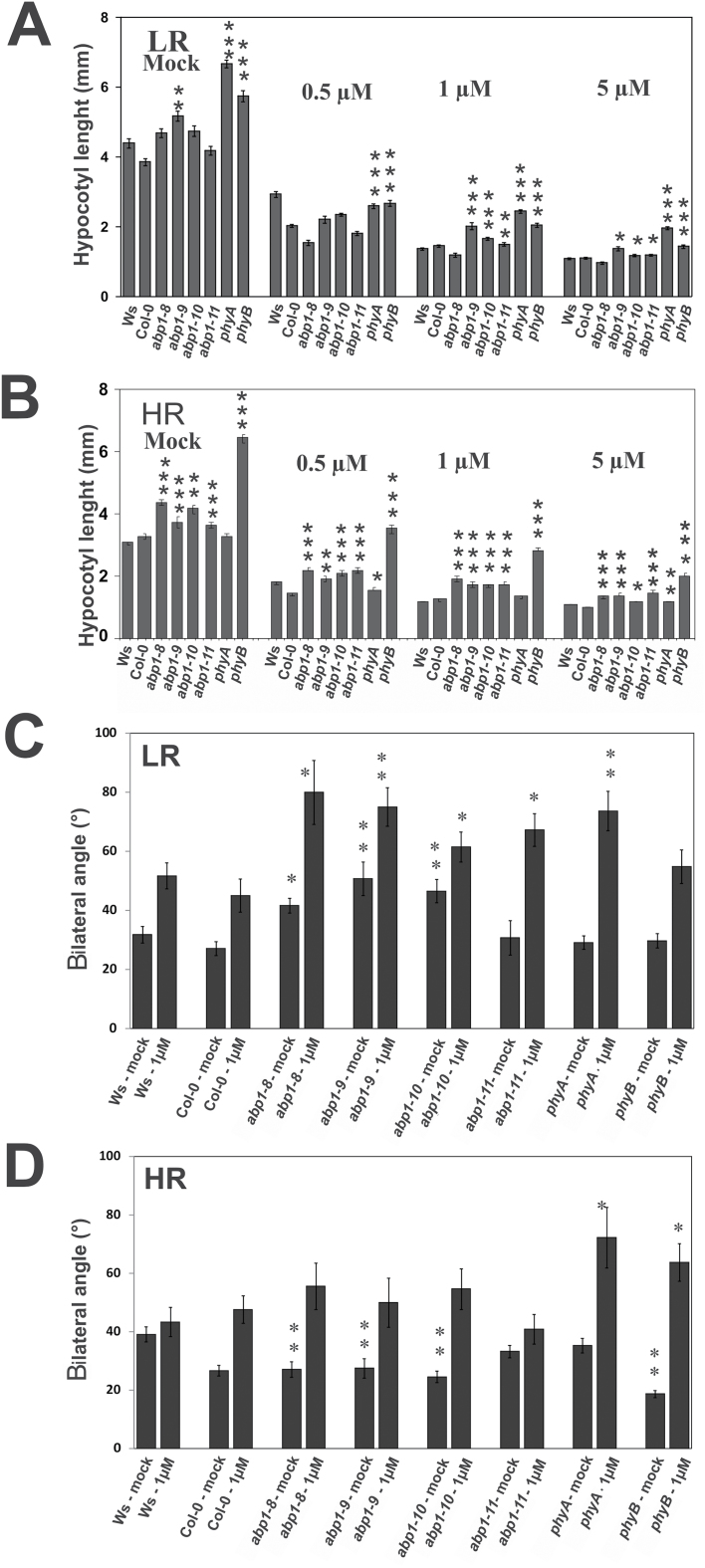
Effect of NPA on elongation and gravitropism in LR or HR. Seedlings were grown for 3 d in W and then for 3 d in low ratio R:FR + W (FR-enriched light) and high ratio R:FR (R-enriched light) on agar without and with indicated NPA concentrations. (A, B) Hypocotyl lengths. (C, D) Absolute values of hypocotyl angles as deviating from the plumb line. Asterisks indicate significances between the wt and mutants: * *P*<0.05; ** *P*<0.01; *** *P*<0.001 mutants (*n*=35 for each genotype; SEM). When error bars of different bars within one experiment do not overlap values are significantly different from each other within one panel.

The elongation response to shade includes regulation of polar auxin transport (Nagashima *et al.*, 2008*a*/*b*). Therefore, we tested the influence of the polar transport inhibitor naphthylpthalamic acid (NPA) on elongation in LR and HR light (Fig. 6A, B). NPA inhibited elongation strongly at 0.5 µM. In LR, *phyB* and more so *phyA* plants were more resistant to NPA than the wt. This property was observed only in *abp1-9* to some extent at 0.5 µM and 1 µM NPA so that an NPA insensitivity was not clearly indicated in *abp1* mutants (Fig. 6A, B). In HR, *phyB* seedlings were clearly more resistant to NPA than all other genotypes (Fig. 6B).

We noticed that inhibition of hypocotyl gravitropism was increased by the combination of either LR or HR with NPA (Nagashima *et al.*, 2008*a*/*b*). In LR *abp1-8*, *abp1-9*, *abp1-10*, but not *abp1-11*, grew less vertical compared with wt (Fig. 6C). This gravitropism inhibition also was the case in the presence of 1 µM NPA for all four *abp1* mutants. In LR alone, *phyA* and *phyB* seedlings responded as the wt, but *phyA* seedlings in LR in the presence of NPA clearly displayed inhibited hypocotyl gravitropism so that the response of the *abp1* mutants was more similar to *phyA* than to *phyB* seedlings.

In HR alone *abp1* seedlings grew more upright than wt seedlings, similar to *phyB* seedlings. This is consistent with a compromised phyB signalling as found in low continuous R (Fig. 5A, B). In HR and added NPA the *abp1* mutants showed a tendency towards inhibited gravitropism but this was not statistically significant as in *phyA* and *phyB* seedlings. LR and HR clearly inhibited gravitropism in *abp1* mutants as in *phyA* and *phyB* seedlings (Fig. 6C, D) (Liscum and Hangarter, 1993; Robson and Smith, 1996). In *abp1-11*, R or FR inhibition of gravitropism was absent, in LR and in the presence of NPA inhibition was apparent. Taking all four sets of data together, inhibition of gravitropism was weakest in *abp1-11* compared with the three *abp1* mutants.

### Expression of light-induced genes in *abp1* mutants

**Fig. 7.**
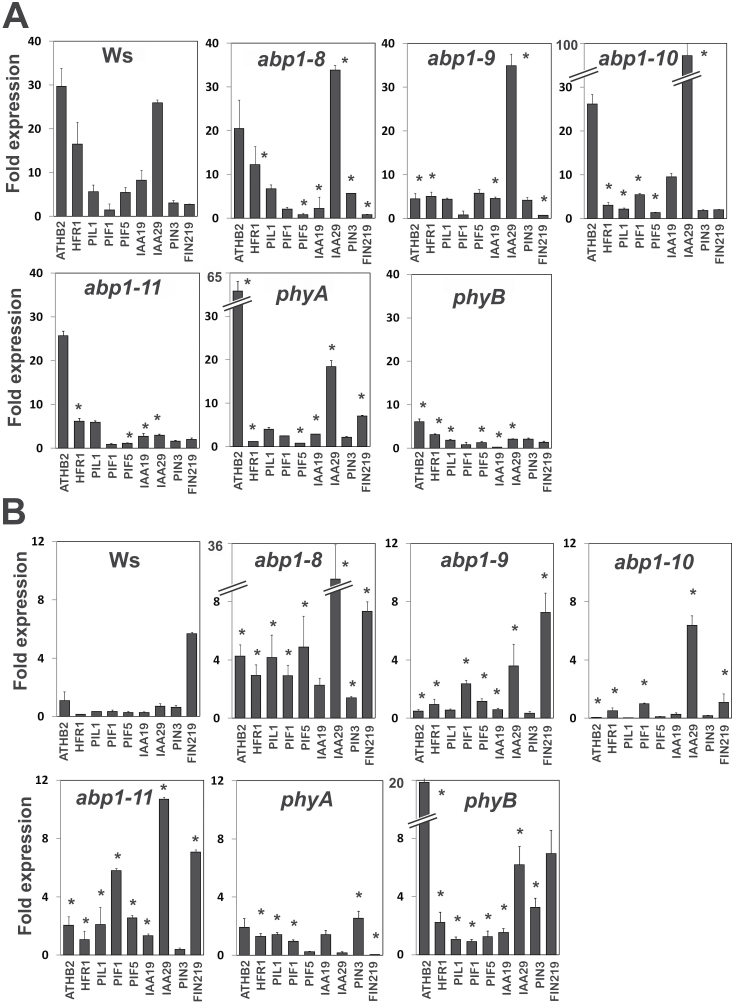
Expression response of shade-induced genes to 1h (A) low ratio R:FR (FR-enriched light) or (B) high ratio R:FR (R-enriched light). Seedlings were grown for 3 d in white light. qPCR data were obtained from at least three biological replications with three technical replications for each gene target. Statistical analysis was as described (Livak and Schmittgen, 2001; Pfaffl *et al.*, 2002). At t=0min fold expression was set as 1 for each genotype (not on the graph). Values are means with SEM (* *P*<0.05).

We investigated expression of ten shade-induced genes (*ATHB2*, *HFR1*, *PIL1*, *PIF1*, *PIF5*, *IAA19*, *IAA29*, *PIN3*, *FIN219*; Fig. 7). References for primers are in Supplementary Table S2. We restricted FR or R light to a short induction period of 1h in W (Wang *et al.* 2011).

In LR, phytochrome mutants and all *abp1* mutants clearly showed a different expression of shade-induced genes than wt. In *phyA*, being wt with respect to *phyB*, high induction of *IAA29* similar to wt was found, and this was also found in *abp1-8*, *abp1-9* and *abp1-10* but not in *phyB* and *abp1-11*. *HFR1* was repressed both in *phyA*, *phyB* and in the *abp1* mutants. *ATHB2* was de-repressed in *phyA* and *abp1-8*, *abp1-10*, and *abp1-11*, but not in *phyB* and *abp1-9*. In *phyB* all test genes were repressed in comparison to the wt. Overall, expression of shade marker genes in *abp1* mutants was, in general, more similar to *phyA* than to *phyB* in LR.

In HR and with *phyB* eight out of nine genes tested were more strongly de-repressed than in the wt, whereas in *phyA* only five genes were de-repressed but generally less than in *phyB*. *FIN219*, a phyA-dependent gene (Wang *et al.*, 2011), was repressed in *phyA*. In *abp1* mutants several genes (4–8) were de-repressed, and therefore in HR they were clearly more similar to *phyB* than to *phyA*.

### Expression of shade-induced and auxin-induced genes in *phyB* seedlings

**Fig. 8.**
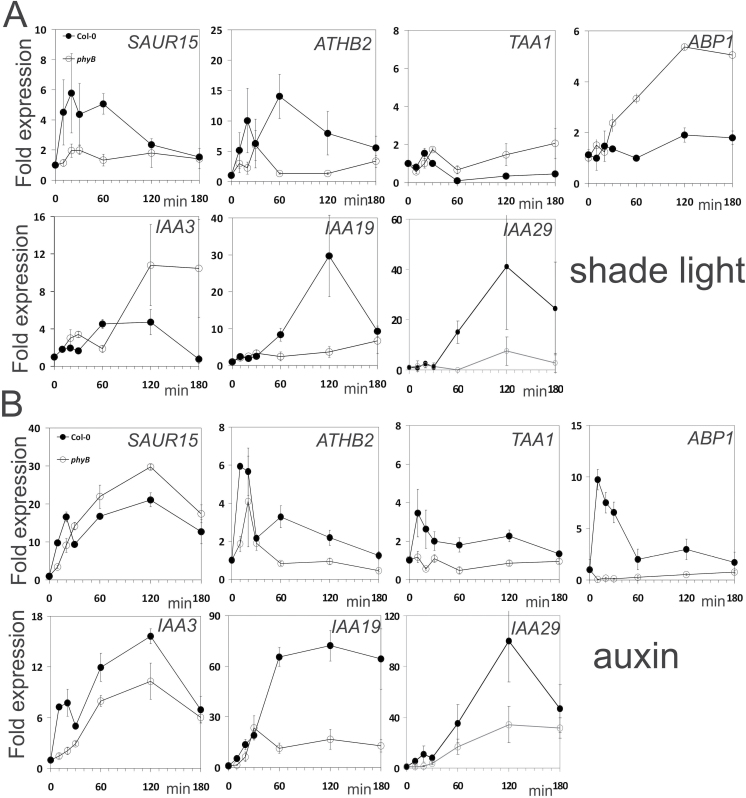
Differential co-regulation of expression of auxin- and light-induced genes in *phyB* and Col wt seedlings. Seedlings were grown for 3 d in w light and then treated in (A) with additional FR in LR light or (B) treated with 10 µM IAA for the times indicated in the graphs. Black symbols: Col; white symbols: *phyB*. qPCR data were obtained from two biological replications with three technical replications for each gene target. Statistical analysis was as described (Livak and Schmittgen, 2001; Pfaffl *et al.*, 2002). At t=0min fold expression was set as 1 for each genotype. Values within one graph are significantly different when error bars or symbols do not overlap (*P*<0.05).

The expression of a number of genes (*IAA3*, *IAA19*, *IAA19*, *SAUR15*, *ATHB2*, *FIN219*) is co-regulated by auxin and shade (Steindler *et al.*, 1999; Devlin *et al.*, 2003; Kunihiro *et al.*, 2011; Leivar *et al.*, 2012) and we included the auxin biosynthesis and shade-induced gene, *TAA1* (Tao *et al.*, 2008) and ABP1 (Effendi *et al*., 2011 2013) into the analysis. We showed that *abp1* mutants misregulate expression of auxin-regulated genes after 10–30min (Fig. 3) and of light-regulated genes after 1h (Fig. 7). However, little is known whether *phyB* seedlings misregulate the expression of auxin-induced genes. Therefore, we investigated the kinetics of induction of these genes by auxin or shade in *phyB* seedlings (Fig. 8).

In the wt (Col), shade induced a rise in expression of *SAUR15*, *ATHB2*, *IAA3*, *and IAA19* and weakly in *IAA29* at 10–20min. The responses peaked at 1–2h with a tendency to decline at 3h (Fig. 8A). Expression of *ABP1* and *TAA1* stayed low in wt and *phyB* seedlings. In *phyB* seedlings, shade-induced expression of *ABP1*, *TAA1*, and *IAA3* was higher than in wt seedlings suggesting that phyB represses these genes. All time courses were fast which indicates a rather direct signal pathway from phyB to its target genes.

We quantified auxin-induced expression in wt seedlings (Fig. 8B). As expected, all genes were induced, although *TAA1* only at a low level. Again, expression started to rise at 10–20min, peaked and declined towards 3h. In *phyB* seedlings, expression of most markers was lower than in wt seedlings; only *SAUR15* was higher in *phyB* than in wt and, surprisingly, expression of *ABP1* was not elevated by auxin in *phyB*. Hence, the *phyB* mutant had a partially aberrant auxin physiology with respect to the expression of marker the genes used here.

## Discussion

### Genetic engineering of stable mutant alleles of *ABP1* by complementation

There is only one *ABP1* gene in the *Arabidopsis* genome (Chen *et al.*, 2001b). The embryo lethality of the T-insertion mutant and the still largely unsuccessful attempts to search for other types of mutants (Braun *et al.*, 2008; Robert *et al.*, 2010; Xu *et al.*, 2010; Effendi *et al.*, 2011) prompted us to engineer strong point mutation alleles by complementation of the knockout to aid investigation of ABP1 functions.

### The novel *abp1* mutants are loss-of-function

Modelling of the mutated binding sites showed that the protein surface contacting 1-NAA is distorted in all mutants (Fig. 1 and Supplementary Table S1). The thermodynamic surface description of the binding pockets and calculated binding energies for the wt and the *abp1* mutants provided an explanation for why we could obtain only a few mutant alleles and why all were loss-of-function mutants.

The mutated and the wt protein in all four complementation lines is tagged, but upstream of the ER retention signal KDEL. Alterations to KDEL and additions like GFP have substantial effects on the exocytosis/endocytosis balance (Robert *et al.*, 2010; Wang *et al.*, 2013). Thus, an important aspect of our lines is that they are characterized in a wide variety of experiments and *abp1-11*, expressing the tagged wt ABP1, is phenotypically similar to wt. Owing to the immense difficulties associated with isolation of these mutants we isolated only one line per genotype so we cannot fully exclude positional effects of the new cDNAs on the respective phenotypes of the four mutants.

The C-terminus and thus its tag protrudes from the protein (Woo *et al.*, 2002) and therefore can be expected to negatively interfere in *abp1-11* with the interaction to essential partners, such as with the four recently identified receptor kinases (Dai *et al.*, 2013; Xu *et al.*, 2014). This notion is supported by the finding that the C-terminus may be mobile and participates in its signalling function (Thiel *et al.*, 1993). Effects originating from overexpression of the mutated *ABP1* cDNA are less likely because the overexpression level reached only1.5–1.7-fold in the mutants and 1.3-fold in *abp1-11* as compared with the wt (Fig. 2A). Moreover, expression of 50% ABP1 in *ABP1*/*abp1* (Fig. 2A) can cause a phenotype very similar to the one observed here in the point mutants (Effendi *et al.*, 2011) so that overexpression is an unlikely cause for the mild phenotype in *abp1-11*.

A near-wild-type phenotype was recorded for *abp1-11* in induction of lateral roots by IAA, phototropism, hypocotyl gravitropism, root gravitropism and lobe formation (Fig. 2). Auxin-induced elongation growth in the dark was dependent on the presence of wt ABP1 in *abp1-11* but was not found in the other three mutants (Fig. 5M). Delay of marker gene expression was apparent but quantitatively lowest in *abp1-11* as compared with *abp1-8*, *abp1-9*, or *abp1-10* (Fig. 3). The permanently high expression of *PIN2* and *PIN3* in all lines (Fig. 3B) may relate to disturbances in tropisms (Petrášek and Friml, 2009) and the response to shade (Keuskamp *et al.*, 2010). In *abp1-8* this increased expression of *PIN2* was not found and *PIN3* expression was lowest as compared with the wt. This could provide an explanation for the similarity of auxin transport in *abp1-8* and the wt (Fig. 2I). In response to various light conditions, *abp1-11* had a light-related phenotype weaker than the other three mutants: in hypocotyl length in W light (Fig. 2B); leaf blade shape (Fig. 4A–D), flowering time (Fig. 4E–G), and hypocotyl elongation in response to high ratio R:FR+W (Fig. 5K) and constant B (Fig. 5F); and increase of the bilateral angle in low ratio R:FR (Fig. 6C) and high ratio R:FR (Fig. 6D) in the presence of 1 µM NPA.

### Auxin phenotypes in *abp1* mutants are linked to auxin transport

Most auxin actions are interwoven with changes in polar auxin transport (Petrášek and Friml, 2009). Here (Fig. 2) and in *ABP1*/*abp1* (Effendi *et al.*, 2011) we showed that basipetal auxin transport in root tips of *abp1* mutants was delayed. Functions such as lateral root formation, tropisms (Petrášek and Friml, 2009), and emergence and growth of epidermal cell lobes (Xu *et al.*, 2010) were all affected in *abp1* mutants and are all dependent on polar auxin transport, supporting the suggestion that ABP1 function is linked to auxin transport-dependent functions.

**Fig. 9.**
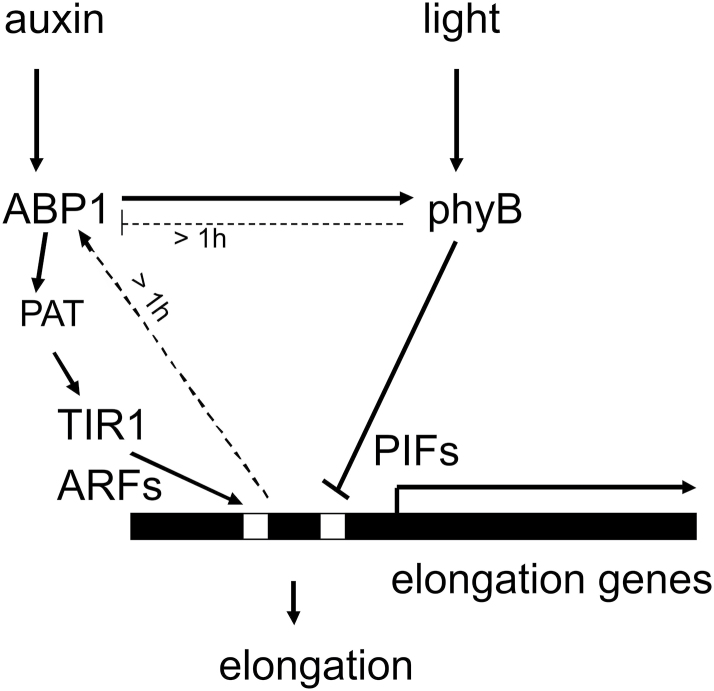
Model of suggested linkages between the receptors ABP1 and phyB and early downstream responses. Only responses during the first hour of stimulus are depicted. Solid arrows indicate functional links without implying a detailed mechanism. Dotted arrows indicate slow transcriptional regulation. Auxin short term responses include the regulation of polar auxin transport (PAT) by ABP1 (Robert *et al.*, 2010) and the influence on TIR1 by the cytosolic auxin concentration.

ABP1 at the apoplastic side of the plasma membrane and the ER lumen cannot directly interact with TIR1 in the nucleus, yet ABP1 is necessary for efficient stimulus–response coupling between the two receptors within 10min (Figs 3 and 8). Four transmembrane receptor kinases were recently found to bind to ABP1 and provide a mechanism for the long sought transmembrane signalling (Dai *et al.*, 2013; Xu *et al.*, 2014). Auxin-induced expression of *ABP1* is detected after 10min (compare Fig. 8B and Effendi *et al.*, 2011; Effendi and Scherer, 2011), but a secreted protein needs roughly 1h to reach the plasma membrane (Scherer, 2011) so that TIR1 cannot regulate the presence or activity of ABP1 in less than 1h (Fig. 9). Short-term effects of NPA inhibition showed down-regulation of PIN protein activity, which consequently would lead to an increased auxin concentration in the cytosol (Covanová *et al.*, 2013) with the logical consequence of re-quantifying TIR1/AFB-dependent transcriptional abundance of auxin marker genes (Scherer *et al.*, 2012).

### ABP1 and phytochrome regulate growth in a tight functional interaction

The most significant result of this study was the compromised R light signalling in *abp1* mutants, although this was indicated in a weaker fashion in *abp1-5* and *ABP1*/*abp1* (Effendi *et al.*, 2013). In brief, *abp1* mutants are compromised in a number of phyB functions (elongation in R, apical dominance, early flowering, inhibition of gravitropism, misregulation of shade marker genes in LR similar to a *phyB* mutant), and some phyA functions (broad leaves, elongation in FR, inhibition of gravitropism, misregulation of shade marker genes in HR similar to a *phyA* mutant).

Interaction between auxin and light in plant growth regulation has been intensively investigated, particularly in responses to shade light (Ruberti *et al.*, 2012). Hypocotyl elongation of the *abp1* mutants was partially insensitive to both continuous FR and R (Fig. 5). Insensitivity to B was also observed and could be a consequence of compromised phyA signalling (Fankhauser and Casal, 2004). ABP1 seems to have a dual role, repression of elongation in the light in conjunction with phytochromes, but supporting elongation in the dark (Fig. 5L, M), which offers an explanation for use of etiolated tissue in the classical auxin growth test. In line with the observations on gravitropism by others (Nagashima *et al.*, 2008a/b), compromised phytochrome signalling is indicated by our observations on effects of R and FR light in conjunction with NPA on gravitropism (Fig. 6).

Our data indicate that ABP1 can crosstalk with phyB and phyA, even though these are located in the cytosol and nucleus. Our short-term marker gene expression experiments provide a basis to understand how to link the responses to their receptors (Figs 3, 7, 8, model in Fig. 9). The advantage of short-term kinetics is that negative or positive back-coupling responses can be minimized. In phenotypic assays the final outcome is the sum of many events over time. Thus, even though regulation of auxin-induced expression of auxin marker genes is executed by TIR1 (Mockaitis and Estelle, 2008), their delayed expression in *abp1* mutants is observed after only 10min (Fig. 3). Therefore, ABP1 for this response acts functionally upstream of TIR1 and exerts a strong influence.

That changes in the status of phyB have such early consequences for auxin signalling was unexpected, and seems to integrate phyB into auxin signalling (see also Reddy and Finlayson, 2014). Expression of marker genes under R or FR control using phytochromes as sensors was altered in *abp1* mutants after 1h (Fig. 7). Therefore mutations in the ABP1 auxin receptor change the light-induced expression of shade marker genes. This argues either for a (i) parallel co-regulation of marker gene expression by auxin-dependent and light-dependent transcription factors or (ii) for a change of the phyB activity status induced by the mutated ABP1, or both (Fig. 9).

For the co-regulation of expression of elongation genes, phyB acts as a repressor activated by R (Steindler *et al.*, 1999; Devlin *et al.*, 2003; Tao *et al.*, 2008; Kunihiro *et al.*, 2011; Leivar *et al.*, 2012). Potential mechanisms of one receptor to regulate the activity of the other are not clear. Because shade does not up-regulate *ABP1* expression in the wt (Fig. 8A), the possibility of positive modulation of ABP1 activity by phyB can be excluded. Rather in the *phyB* mutant *ABP1* transcription was slowly increased in shade light, not in the wt. This indicates that a long-term inhibition of ABP1 protein activity by phyB is a possibility (Fig. 9).

Could ABP1 regulate phyB activity? Signalling from ABP1 to phytochromes could start with ABP1 interaction with a transmembrane co-receptor that has the capacity to modulate the phyB phosphorylation status (Effendi *et al.*, 2013; Medzihradszky *et al.*, 2013; Nito *et al.*, 2013; Xu *et al.*, 2014). This speculative mechanism could explain how an auxin transmembrane signal to a network of cytosolic proteins could be transmitted.

The physiology of hypocotyl elongation in our *abp1* mutants is fully compatible with this model. Elongation in the dark is ABP1-modulated (Fig. 5M) and repressed by phyB in the light. IAA cannot overcome this light repression (Fig. 5L). Shade releases the repression (Fig. 5I–K) by inactivation of phyB. Partial R and FR insensitivity of elongation in *abp1* mutants (Fig. 5A–H) is also consistent with this model in that ABP1 supports the action of phyB in light and repression of phyB action is weakened in an *abp1* mutant.

Auxin transport and auxin biosynthesis were suggested to play a role in shade-induced elongation (Nagashima *et al.*, 2008a/b; Tao *et al.*, 2008), which is not mutually exclusive for the functions discussed above. The effects of NPA on elongation and on gravitropism in LR and HR (Fig. 6) are consistent with the concept of weakened phyB action. Regulation of auxin transport in shade was suggested to depend on PIN efflux facilitators (Keuskamp *et al.*, 2010) and ABCB efflux transporters (Nagashima *et al.*, 2008a/b), the latter being shown to be directly inhibited by NPA (Bailly *et al.*, 2012).

Ordering early responses into a timeline as a first strategy (Figs 3, 7, 8) is a way to mechanistically explain the functional interaction of ABP1 and phytochromes (Fig. 9) and provides a fresh starting point to investigate auxin and R signalling and growth control.

## Author contributions

YE performed and designed experiments, NF did quantum modelling, MG did transport experiments, CL performed transcription experiments, GFES designed the research and GFES, YE, NF, and MG wrote the paper.

## Supplementary Material

Supplementary Data
