# Supplementary material for: Complementation of the embryo-lethal T-DNA insertion mutant of AUXIN-BINDING-PROTEIN 1 (ABP1) with abp1 point mutated versions reveals crosstalk of ABP1 and phytochromes
Source: J Exp Bot. 2014 Nov 11;66(1):403–18. doi: 10.1093/jxb/eru433 (PMC4265171; doi:10.1093/jxb/eru433)
Supplement: Supplementary Data [file supp_66_1_403__index.html]

Complementation of the embryo-lethal T-DNA insertion mutant of AUXIN-BINDING-PROTEIN 1 (ABP1) with abp1 point mutated versions reveals crosstalk of ABP1 and phytochromes — Complementation of the embryo-lethal T-DNA insertion mutant of AUXIN-BINDING-PROTEIN 1 (ABP1) with abp1 point mutated versions reveals crosstalk of ABP1 and phytochromes — Supplementary Data 

# Complementation of the embryo-lethal T-DNA insertion mutant of *AUXIN-BINDING-PROTEIN 1* (*ABP1*) with *abp1* point mutated versions reveals crosstalk of ABP1 and phytochromes

## Supplementary Data

Data files

**Files in this Data Supplement:**

- Supplementary Data - Supplementary Data
