# Supplementary material for: Complementation of the embryo-lethal T-DNA insertion mutant of AUXIN-BINDING-PROTEIN 1 (ABP1) with abp1 point mutated versions reveals crosstalk of ABP1 and phytochromes
Source: J Exp Bot. 2014 Nov 11;66(1):403–18. doi: 10.1093/jxb/eru433 (PMC4265171; doi:10.1093/jxb/eru433)
Supplement: Supplementary Data [file supp_eru433_jexbot132449_file001.pdf]

Supplemental Figures and Tables

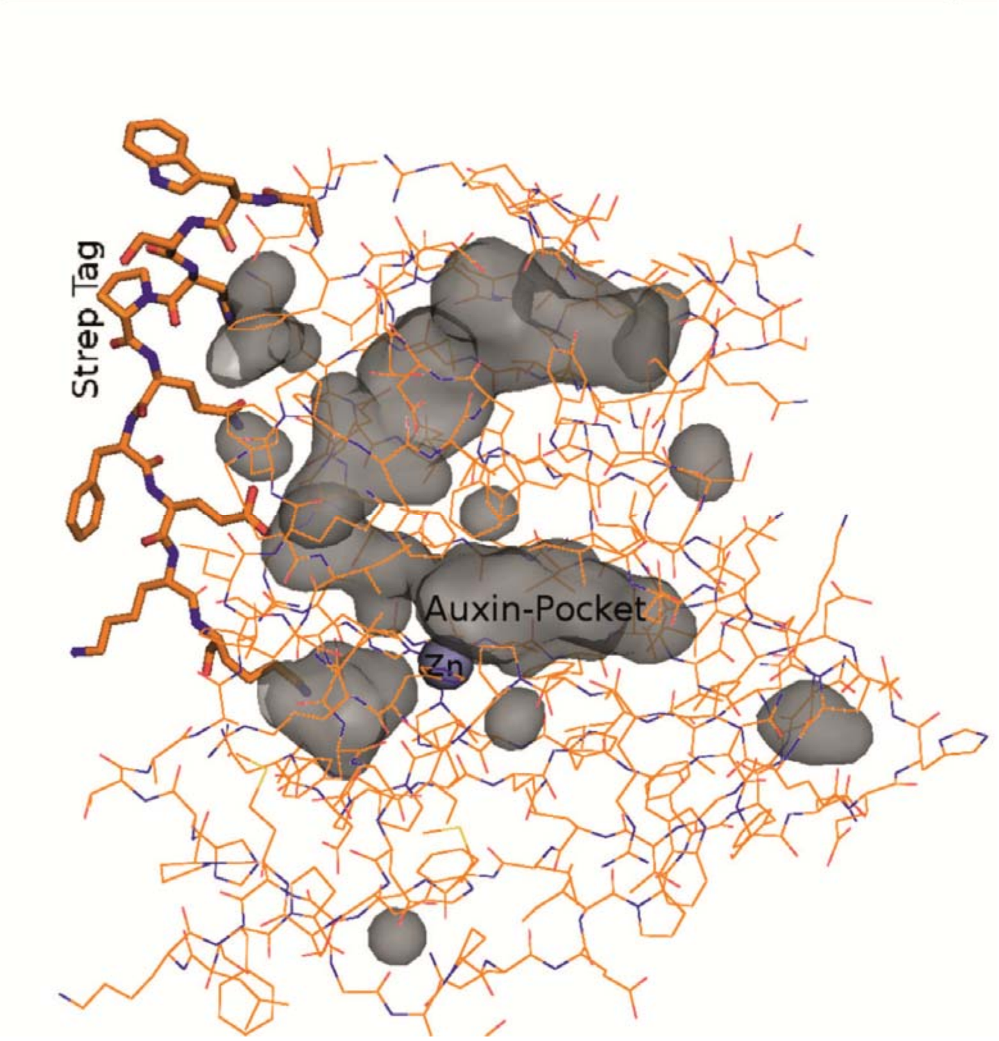

Supplemental Figure 1. 3-dimensional structure of the strep tag attached at the C-terminus of ABP1. Gray areas are all hollow spaces in the ABP1 protein which could accommodate ligands. The potential access channel for auxin is indicated and the auxin binding pocket. The large gray area in the upper part of the figure could harbor small molecules.

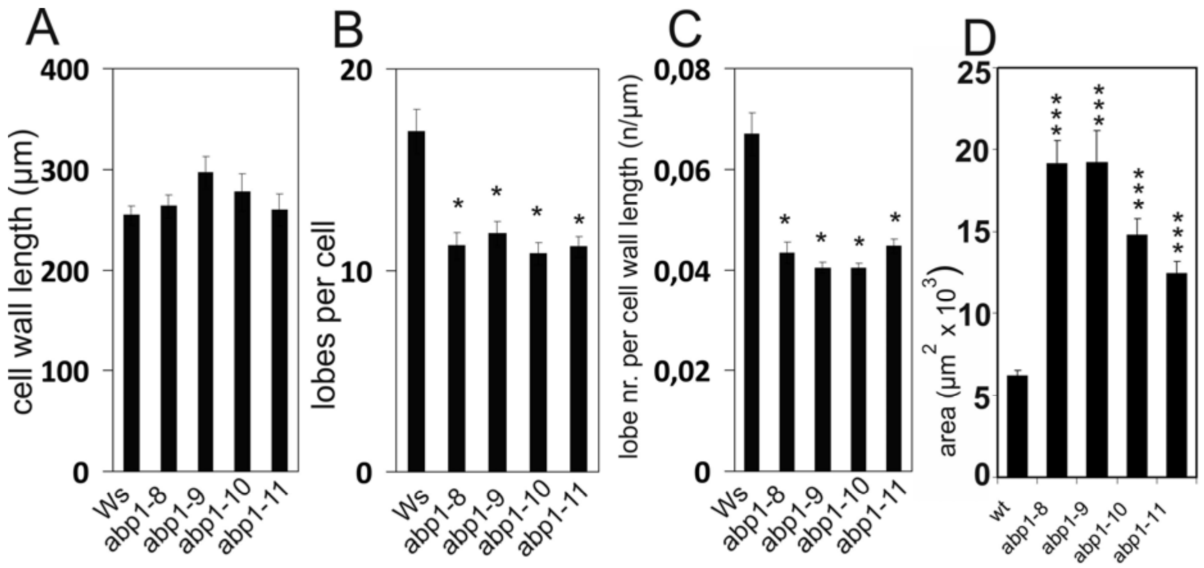

Supplemental Figure 2. Quantification of lobe formation in *abp1* mutants. (A) Length of cell walls of epidermal cells. (B) Number of lobes per cell. (C) Lobes per length of circumference. (D) Epidermal cell areas. (n>20; S.E.; *= p<0.05; ***=p<0.001).

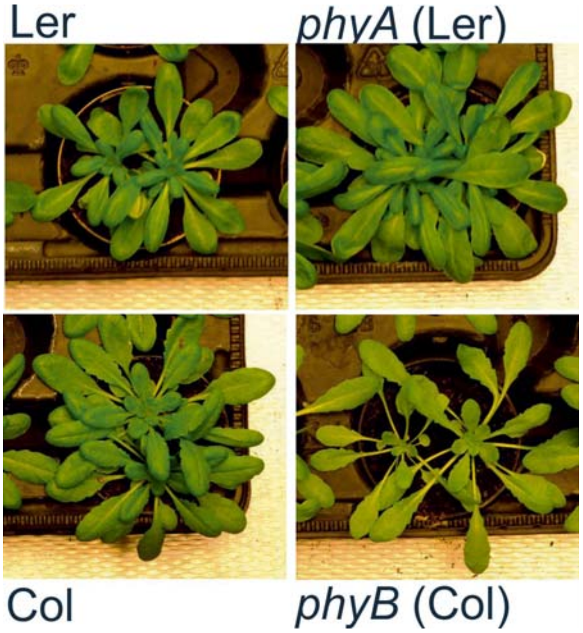

Supplemental Figure 3. Rosettes of *phyA* and *phyB* mutants grown side by side with respective wt plants. Shown were plants grown for 55 d in short days (8h/16h).

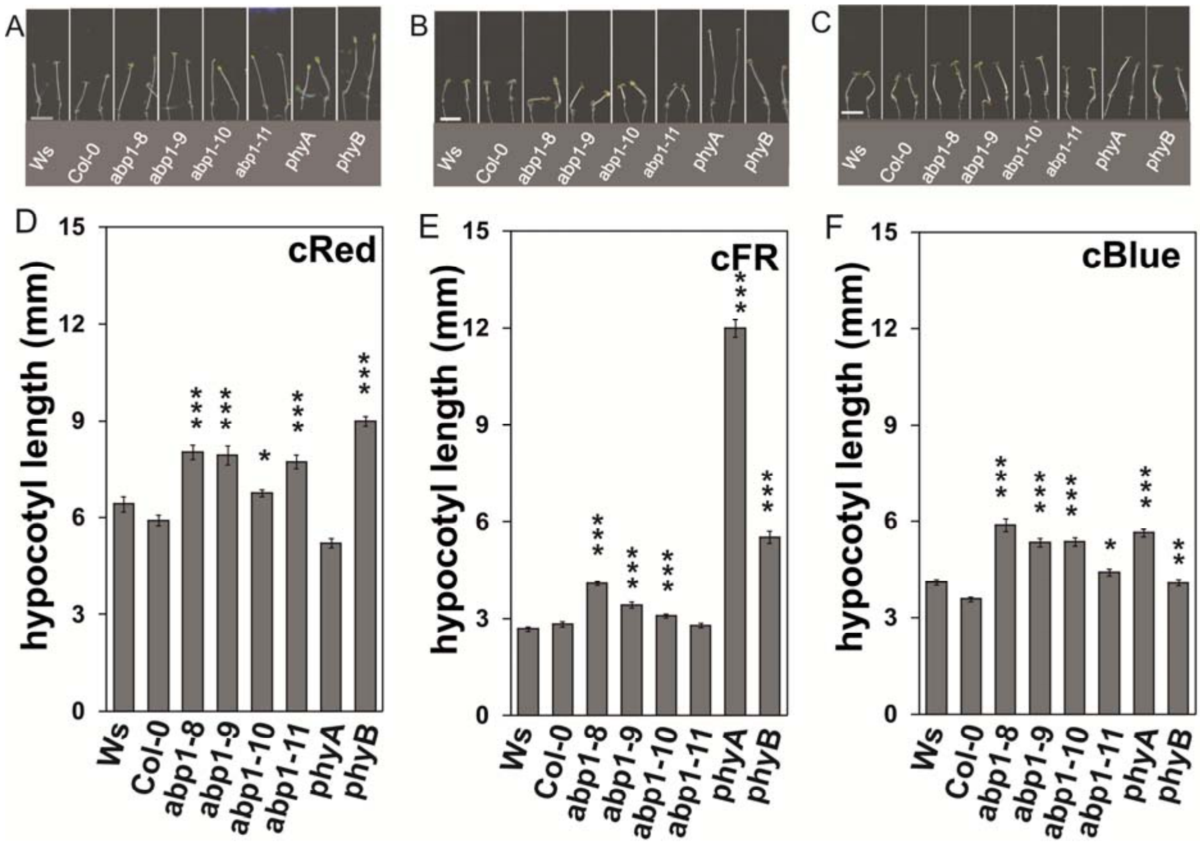

Supplemental Figure 4. Elongation in monochromatic continuous light (R, FR, B) of 4 d old seedlings (1 µmol m^{-1} s^{-2} each). Seedlings were grown for four days. (A-C) Representative images of seedlings grown in FR, R, B respectively. (D-F) Hypocotyl lengths (n>80; S.E.; *: p<0.05;**: p<0.01; ***: p<0.001).

Supplemental Table 1. Quantum chemical modeling of auxin binding pocket in the wt and several *abp1* mutants.

dE energies resemble binding energies, the auto values of the Overlap (representing electron density of he binding box) and Coulomb (representing the charge surface of the binding box) matrix are given in the second and third row, respectively. In the bottom part of the table, for clarity, only the differences are presented. When comparing different mutations the differences of the surface charges (Coulomb) and the electron density (Overlap) of the box rather than the total values are important. The three values together have a strong influence on the biological efficacy. (ΔE (Kcal/mol); $X^{wt} = X - WT$).

	**wt**	**H106>N106**	**T25>Y25**	**L54>I54**	**W151>A151**
		abp1-10	*abp1-9*	*abp1-8*	not realized
dE(Kcal/mol)	-41.10	-37.51	-42.14	-35.90	-37.56
Overlap	13740.29	13705.01	13918.43	13722.70	13430.28
Coulomb	111916.90	109955.50	115202.40	112869.60	101709.00
dE^{wt} (Kcal/mol)	0.00	3.59	-1.04	5.20	3.53
$Overlap^{wt}$	0.00	-35.28	178.14	-17.59	-310.01
$Coulomb^{wt}$	0.00	-1961.40	3285.50	952.70	-10207.90

Supplemental Table 3. Primers for PCR

Auxin treatment:

18S rRNA forw	5'-GGC TCG AAG ACG ATC AGA TAC C-3';
18S rRNA rev	5'-TCG GCA TCG TTT ATG GTT-3';
ABP1 forw	5'-ACG AGA AAA TCA TAC CAA TTC GGA CTA ACC-3';
ABP1 rev	5'-GTA TCT ACG TAG TGT CAC AAA ACC TCA AC-3';
IAA2 forw	5'-GGT TGG CCA CCA GTG AGA TC-3';
IAA2 rev	5'-AGC TCC GTC CAT ACT CAC TTT CA-3';
IAA3 forw	5'- CAAAGATGGTGATTGGATGCT-3';
IAA3 rev	5'- TGATCCTTAGTCTCTTGCACGTA-3';
IAA11 forw	5'-CCT CCC TTC CCT CAC AAT CA-3';
IAA11 rev	5'-AAC CGC CTT CCA TTT TCG A-3';
IAA14 forw	5'-CCT TCT AAG CCT CCT GCT AAA GCA C-3';
IAA14 rev	5'-CCA TCC ATG GAA ACC TTC AC-3';
IAA19 forw	5'-GGT GAC AAC TGC GAA TAC GTT ACC-3';
IAA19 rev	5'-CCC GGT AGC ATC CGA TCT TTT CA-3';
IAA20 forw	5'-CAATATTTCAACGGTGGCTATGG-3';
IAA20 rev	5'-GCC ACA TAT TCC GCA TCC TCT A-3';
GH3.5 forw	5'-AGC CCT AAC GAG ACC ATC CT-3;
GH3.5 rev	5'-AAG CCA TGG ATG GTA TGA GC-3';
SAUR9 forw	5'-GAC GTG CCA AAA GGT CAC TT-3';
SAUR9 rev	5'-AGT GAG ACC CAT CTC GTG CT-3';
SAUR15 forw	5'-ATG GCT TTT TTG AGG AGT TTC TTG GG-3;
SAUR15 rev	5'-TCA TTG TAT CTG AGA TGT GAC TGT G-3';

SAUR23 forw	5'-ATG GCT TTG GTG AGA AGT CTA TTG GT-3;
SAUR23 rev	5'-TCA ATG GAG CCG AGA AGT CAC ATT GA-3'.
PIN1-forw	5'GGA GAC TTA AGT AGG AGC TCA GCA-3';
PIN1-rev	5'-CCA AAA GAG GAA ACA CGA ATG-3';
PIN2-forw	5'-TAT CAA CAC TGC CTA ACA CG-3';
PIN2-rev	5'-GAA GAG ATC ATT GAT GAG GC-3';
PIN3-forw	5'-GAG TTA CCC GAA CCT AAT CA-3';
PIN3-rev	5'-TTA CTG CGT GTC GCT ATA GT-3';
PIN5-forw	5'-ACC CTG CCG CTC TTC ACC A-3';
PIN5-rev	5'-GCC CAC AAC GCT AAG ACC G-3';

Light experiments:

*ATHB2*_F	5'-GAG GTA GAC TGC GAG TTC TTA CG 3'
*ATHB2*_F	5'-GCA TGT AGA ACT GAG GAG AGA GC-3'
*HFR1*_F	5'-CAC AAG ACG GAC AAG GTT TCG-3'
*HFR1*_R	5'-GTC AGC ATG TGG TTG TGC ATT C-3'
*PIL1*_F	5'-TGG TGC CTT CGT GTG TTT CTC A-3'
*PIL1*_R	5'-GGA CGC AGA CTT TGG GAA TTG-3'
*PIF5*_F	5'-GAT GCA GAC CGT GCA ACA AC-3'
*PIF5*_R	5'-CTT TTA TGC TTG CTT AGG CG-3'
*PIF1*_F	5'- CCCGTCAAGAGTCTTTGTACC-3'
*PIF1*_R	5'- CCCGAGGTTGGATCATACTG-3'
*IAA29*_F	5'- TCCTCTGGAATCCGAGTCTTC-3'
*IAA29*_R	5'- GGTGGCCATCCAACAACTT-3'
*IAA19*_F	5'-GGT GAC AAC TGC GAA TAC GTT ACC-3';
*IAA19*_R	5'-CCC GGT AGC ATC CGA TCT TTT CA-3';
*PIN3*_F	5'-GAG TTA CCC GAA CCT AAT CA-3';
*PIN3*_R	5'-TTA CTG CGT GTC GCT ATA GT-3';
*FIN219*_F	5'- GTCATCACAAATTACGCAGGGTTG-3'
*FIN219*_R	5'- TCTCTTTCGGTGTTCTTGTCGATG-3'
*TAA1*_F	5'-TGG ATC ATG GTG ATC CAA CG-3'
*TAA1*_R	5'-GCT CAA GCA ACC AAC ACA AG-3'

PCR genotyping was done using reverse ABP1 genomic primer (5'-CCT GAG ATC TCA AGT AGG AAG CGT C-3') and right border primer (5'-TCC CAA CAG TTG CGC ACC TGA ATG-3') primer (Chen et al., 2001b).

Literature describing the shade marker genes:

Carabelli, M., Morelli, G., Whitelam, G., and Ruberti, I. (1996). Twilight-zone and canopy shade induction of the *ATHB-2* homeobox gene in green plants. Proc. Natl. Acad. Sci. USA **93:** 3530-3535.

Carabelli, M., Sessa, G., Baima, S., Morelli, G., Ruberti, I. (1993). The *Arabidopsis Athb-2* and *-4* genes are strongly induced by far-red-rich light. Plant J. **4:** 469-479.

Devlin, P., Yanovsky, M.J., and Kay, S.A. (2003). A genomic analysis of the shade avoidance response in *Arabidopsis*. Plant Physiol. 133: 1617-1629.

Hornitschek, P., Kohnen, M.V., Lorrain, S., Rougemont, J., Ljung, K., López-Vidriero, I., Franco-Zorrilla, J.M., Solano, R., Trevisan, M., Pradervand, S., Xenarios, I., and Fankhauser, C. (2012). Phytochrome interacting factors 4 and 5 control seedling growth in changing light conditions by directly controlling auxin signaling. Plant J. **71:** 699-711.

Hornitschek, P., Lorrain, S., Zoete, V., Michielin, O., and Fankhauser, C. (2009). Inhibition of the shade avoidance response by formation of non-DNA binding bHLH heterodimers. EMBO J. **28:** 3893-3902.

Hsieh, H.L., Okamoto, H., Wang, M., Ang, L.H., Matsui, M., Goodman, H., and Deng, H.W. (2000). *FIN219*, an auxin regulated gene, defines a link between phytochrome A and the downstream regulator COP1 in light control of *Arabidopsis* development. Genes Dev. **14:** 1958–1970.

Keuskamp, D.H., Pollmann, S., Voesenek, L.A.C.J., Peeters, A.J.M., and Pierik, R. (2010). Auxin transport through PIN-FORMED 3 (PIN3) controls shade avoidance and fitness during competition. Proc. Natl. Acad. Sci. USA **107:** 22740-22744.

Kunihiro, A., Yamashino, T., Nakamichi, N., Niwa, Y., Nakanishi, H., and Mizuno, T. (2011). Phytochrome-interacting factor 4 and 5 (PIF4 and PIF5) activate the homeobox ATHB2 and auxin-inducible *IAA29* genes in the coincidence mechanism underlying photoperiodic control of plant growth of *Arabidopsis thaliana*. Plant Cell Physiol. **52:** 1315-1329.

Leivar, P., Tepperman, J.M., Cohn, M.M., Monte, E., Al-Sady, B., Erickson, E., and Quail, P.H. (2012). Dynamic antagonism between phytochromes and PIF family basic helix-loop-helix factors induces selective reciprocal responses to light and shade in a rapidly responsive transcriptional network in Arabidopsis. Plant Cell **24:** 1398-1419.

Nozue, K., Harmer, S.L., and Maloof, J.N. (2011). Genomic analysis of circadian clock-, light-, and growth-correlated genes reveals PHYTOCHROME-INTERACTING FACTOR5 as a modulator of auxin signaling in *Arabidopsis*. Plant Physiol. **156:** 357-372.

Roig-Villanova, I., Bou-Torrent, J., Galstyan, A., Carretero-Paule, L., Portolés, S., Rodríguez-Concepción, M., and Martínez-García, J.F. (2007). Interaction of shade avoidance and auxin responses: a role for two novel atypical bHLH proteins. EMBO J. **26:** 4756-4767.

Ruberti, I., Sessa G., Ciolfi, A., Possenti, M., Carabelli, M., and Morelli, G. (2012). Plant adaptation to dynamically changing environment: The shade avoidance response. Biotechnol. Adv. **30:** 1047-1058.

Salter, M.G., Franklin, K.A., and Whitelam, G.C. (2003). Gating of the rapid shade-avoidance response by the circadian clock in plants. Nature **426:** 680-683.

Sessa, G., Carabelli, M., Sassi, M., Ciolfi, A., Possenti, M., Mittempergher, F., Becker, J., Morelli, G., and Ruberti, I. (2005). A dynamic balance between gene activation and repression regulates the shade avoidance response in *Arabidopsis*. Genes Dev. **19:** 2811-2815.

Stamm, P., and Kumar, P.P. (2010). The phytohormone signal network regulating elongation growth during shade avoidance. J. Exp. Bot. **61:** 2889-2903.

Steindler, C., Mateucci, A., Sessa, G., Weimar, T., Ohgishi, M., Aoyama, T., Morelli, G., and Ruberti, I. (1999). Shade avoidance responses are mediated by the ATHB-2 HD-Zip protein, a negative regulator of gene expression. Development **126:** 4235-4245.

Stepanova, A.N., Robertson-Hoyt, J., Yun, J., Benavente, L.M., Xie, D.Y., Dolezal, K., Schlereth, A., Jürgens, G., Alonso, J.M. (2008) **TAA1**-mediated auxin biosynthesis is essential for hormone crosstalk and plant development. Cell 133: 177-191.

Wang, J.G., Chen, C.H., Chien, C.T., and Hsieh, H.L. (2011). FAR-RED **INSENSITIVE219** modulates CONSTITUTIVE PHOTOMORPHOGENIC1 activity via physical interaction to regulate hypocotyl elongation in *Arabidopsis*. Plant Physiol. **156:** 631-646.

Zhou, Z.Y., Zhang, C.G., Wu, L., Zhang, C.G., Chai, J., Wang, M., Jha, A., Jia, P.F., Cui, S.J., Yang, M., Chen, R., Guo, G.Q. (2011) Functional characterization of the CKRC1/TAA1 gene and dissection of hormonal actions in the Arabidopsis root. Plant J. 66: 516-527.
